# Medicinal mushrooms as an attractive new source of natural compounds for future cancer therapy

**DOI:** 10.18632/oncotarget.25660

**Published:** 2018-06-26

**Authors:** Artem Blagodatski, Margarita Yatsunskaya, Valeriia Mikhailova, Vladlena Tiasto, Alexander Kagansky, Vladimir L. Katanaev

**Affiliations:** ^1^ Centre for Genomic and Regenerative Medicine, School of Biomedicine, Far Eastern Federal University, Vladivostok, Russian Federation; ^2^ Department of Pharmacology and Toxicology, University of Lausanne, Lausanne, Switzerland; ^3^ Federal Scientific Center of the East Asia Terrestrial Biodiversity FEB RAS, Vladivostok, Russia

**Keywords:** cancer, fungotherapy, medicinal mushrooms, targeted treatment, biomedicine

## Abstract

Medicinal mushrooms have been used throughout the history of mankind for treatment of various diseases including cancer. Nowadays they have been intensively studied in order to reveal the chemical nature and mechanisms of action of their biomedical capacity. Targeted treatment of cancer, non-harmful for healthy tissues, has become a desired goal in recent decades and compounds of fungal origin provide a vast reservoir of potential innovational drugs. Here, on example of four mushrooms common for use in Asian and Far Eastern folk medicine we demonstrate the complex and multilevel nature of their anticancer potential, basing upon different groups of compounds that can simultaneously target diverse biological processes relevant for cancer treatment, focusing on targeted approaches specific to malignant tissues. We show that some aspects of fungotherapy of tumors are studied relatively well, while others are still waiting to be fully unraveled. We also pay attention to the cancer types that are especially susceptible to the fungal treatments.

## INTRODUCTION

Nature has since long been an important source of inspiration for the medicine. Throughout evolution, nature produces a vast diversity of biologically active substances, which possess enormous therapeutic potential, amongst other things regarding the treatment of cancers. Natural products have already yielded a series of compounds widely used in anticancer chemotherapy, whilst application of such products in folk and traditional medicine has always been an important clue pointing to potential new sources of compounds with therapeutic potential. Well-known examples include camptothecin derived from the bark and stem of the tree *Camptotheca acuminata* used in Chinese traditional medicine [1], vinca alcaloids derived from Madagascan periwinkle [2] or taxanes derived from the Pacific Yew [3]. Nevertheless, such “first-generation” natural chemotherapeutic agents are directed mostly against housekeeping processes (such as DNA replication or microtubule polymerization and stabilization), which are more active against fast proliferating cancer cells, but are in no way cancer-specific. This results in a variety of harmful side effects in the conventional anticancer chemotherapy, up to eventual patient’s death due to overdose. More up-to-date approaches to cancer treatment involve targeted therapies specific to the hallmarks of cancer and harmless or of low harm to the healthy tissues [4]. Search for compounds able to selectively act on cancer cells or on tumorigenic processes is therefore a problem of the highest priority in the field. Thus, it is a task of great importance to “mine the treasury” of natural products for such compounds in order to expand the arsenal of modern oncology with a variety of highly specific tools.

Cancer fungotherapy is a promising scientific field, which deals with antitumor substances derived from mushrooms. It has been an integral part of the world traditional medicine since the antiquity [5].

The concept of fungal treatment officially appeared in Traditional Chinese Medicine and can be dated back to several thousand years ago [6]. The ancient Chinese pharmacopoeia included hundreds of herbal and fungal species - the latter were considered to be the most effective natural remedies for various types of tumors [6]. In other countries of East and Southeast Asia, mushrooms were also highly valued and rated as “beneficial to health” for centuries. Plant and fungal products were also widespread in Russia, representing the main medicinal resources until the 18^th^ century [7].

In the middle of the 20^th^ century, some of the earliest scientific research was performed on *Boletus edulis* in order to study the antitumor activity of edible and medicinal mushrooms [8].

Over the past 60 years, the rate of studies focusing on fungi increased exponentially, but in many areas of research mushrooms as potential source for beneficial products are still ignored. For instance, 90% of fungal species were never analyzed with respect to their antibiotic and antitumor activity. Moreover, a large part of cancer-related investigations done on fungi deals merely with characterization of unspecific cytotoxic or cytostatic effects on cancer cells (such effects would be harmful for healthy cells as well), rather than with modulation of specific oncogenic signaling pathways, which could be targets for modern, highly specific anticancer therapies. Importantly, a tumor has many “weak spots” and can be targeted at different levels, such as tumor-specific pro-proliferation signaling, regulation of apoptosis, cancer-specific metabolism, angiogenesis, metastasis and, last but not least, modulation of the immune system. The peculiarity of medicinal mushrooms is that, being producers of hundreds of compounds, they can affect multiple cancer-related processes in synergistic ways when used as a treatment. Thus, not only studies of certain fungal-derived compounds are important, but also research on complex anticancer effects caused by the combinations of molecules in their extracts is of a high interest.

In this review, we intend to analyze the recent knowledge, potential to cover the abovementioned processes on the example of four Basidiomycota mushrooms: *Fomitopsis pinicola, Hericium erinaceus, Trametes versicolor* and *Inonotus obliquus.* The fields of cancer fungotherapy and of search for novel antitumor agents are by far not limited to these species; however, these four can serve as typical representatives of widespread medicinal mushrooms used both in traditional medicine and in modern biomedical research. They belong to three different orders, and are a rich source of bioactive compounds such as polyphenols, polysaccharides, glucans, terpenoids, steroids, cerebrosides and proteins, which can be used for treatment of various cancers (Table 1). We chose these representatives to convexly illustrate the therapeutic potential of the fungi and fungal-derived products in relation to cancer and to inspire further interdisciplinary work at the junction of oncology and mycology, which should result in future discoveries of novel low-toxic drugs with highly specific antitumor activities.

**Table 1:** The metabolites found in medicinal mushrooms and their therapeutic potential against cancer

Species	Compound/derivative	Targets/mechanisms of action	Cancer types affected	Experimental models	References
*Fomitopsis pinicola*	Methanol extract	Cytotoxicity	Hepatocarcinoma, cervical cancer	Cell lines	[16]
	Chlorophorm extract	ROS-mediated apoptosis	Colorectal cancer	Cell lines	[20]
		Metalloproteinase-mediated migration inhibition	Colorectal cancer	Cell lines	[20]
	Ergosterol	ROS-mediated apoptosis	Colorectal cancer	Cell lines	[20]
	Ethanol extract	Tumor growth arrest	Sarcoma	Mouse xenograft tumors	[21]
*Hericium erinaceus*	Ethanol extract	Tumor growth arrest	Gastric, liver, colon cancer	Cell lines, Mouse xenograft tumors	[29]
	Water extract	Metalloproteinase-mediated migration inhibition, suppression of ERK and JNK kinase activation	Colon carcinoma	Mouse xenograft tumors	[30]
		NK cells and macrophages stimulation, arrest of angiogenesis	Colon carcinoma	Mouse xenograft tumors	[30]
		Apoptosis via downregulation of antiapoptotic proteins	Leukemia	Cell lines	[31]
	Polysaccharides	Immunostimulation,	-	Mouse models	[32-34]
	Erinacine A (ditherpenoid)	ROS-mediated cell cycle arrest	Gastrointestinal cancer, colorectal cancer	Cell lines, Mouse xenograft tumors	[35-37]
		Antiinvasive			
	Cerebroside E	Angiogenesis blocker	-	HUVEC cell line	[38]
	HEP3 protein	Immunostimulation via gut microbiota	Adenocarcinoma	Mouse xenograft tumors	[39]
	HEG-5 glycoprotein	Proapoptotic stimulation	Gastric cancer	Cell lines	[40]
	Ethanol extract	Cytoprotective	Gastric ulcer (carcinogenic condition)	Rat model	[42]
	1-(5-chloro-2-hydroxyphenyl)-3-methyl-1-butanone,2,5-bis(methoxycarbonyl)terephthalic acid	Helicobacter Pylori growth inhibition	Gastric ulcer (carcinogenic condition)	*In vitro* bacterial growth models	[43]
*Inonotus obliquus*	Water extracts	Cytotoxic/cytostatic	Colon cancer, liver cancer	Cell lines	[52-54]
		Tumor growth inhibition	Melanoma	Mouse xenograft tumors	[55]
			Sarcoma		[56]
	Inonotodiol and inonotsuoxides (lanostan-type triterpenoids)	Tumor growth inhibition	Skin cancer, leukemia	Mouse xenograft tumors	[48,57,58]
	Polyphenoles	Topoisomerase II inhibition (growth arrest)	Colon carcinoma	Cell lines	[59]
	3,4-dihydroxybenzalacetone	NF-κB inhibition-mediated apoptosis, suppression of invasion	Gastric, liver, colon cancer	Cell lines	[60]
	Polysaccharides	Tumor growth inhibition via immunostimulation	Colorectal cancer, gastric cancer	Mouse xenograft tumors	[47, 61-63]
		Migration inhibition, anti-metastatic activities	Lung carcinoma	Cell lines	[64,65]
	Ergosterol peroxide	Inhibition of Wnt signaling	Colorectal cancer	Cell lines, Mouse xenograft tumors	[66]
	Inotodiol		Breast cancer. lung cancer	Cell lines Rat model	[68,69]
*Trametes versicolor*	Water-ethanol extract	Cytotoxic/antiproliferative	Breast cancer, cervical cancer, B-lymphoma, hormone-dependent liver cancer	Cell lines	[72,73]
	Ethanol extract	Cytotoxic/antiproliferative	Prostate cancer	Cell lines	[74]
	Glucans	Tumor growth inhibition	Sarcoma	Mouse xenograft tumors	[75]
	β-glucan-based polysaccharopeptide fraction (PSP)	Tumor growth inhibition via immunostimulation	Breast cancer, gastrointestinal cancer, lung cancer	Mouse xenograft tumors, clinical trials	[76-81]
	Polysaccharide fraction known as Krestin (PSK)				[76, 82-91]
	YZP protein	Immunostimulation	Pancreatic cancer	Cell lines, Mouse xenograft tumors	[70]

### Fomitopsis pinicola

**Figure 1:**
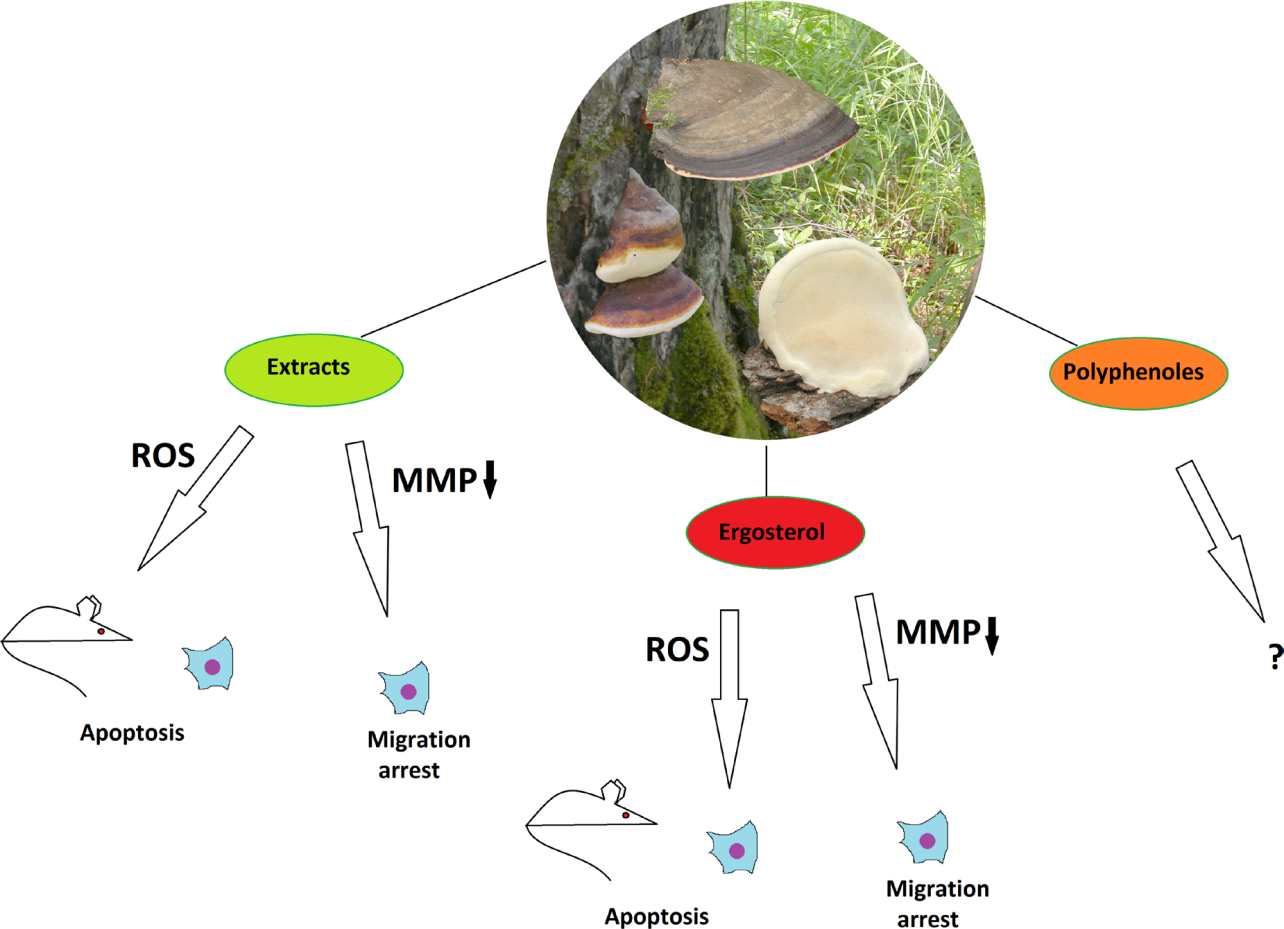
The anticancer properties of *Fomitopsis pinicola* Effects of different mushroom derivatives and their mechanisms of actions on various models are depicted. Mouse and cell icons indicate results obtained on animal and cell models, respectively. ROS – reactive oxygen species, MMP↓ – downregulation of matrix metalloproteinases.

*Fomitopsis pinicola*, class Agaricomycetes, order Polyporales, family Fomitopsidaceae (common name Red Belted Conk) (Figure 1), is a brown rot fungus, a member of Basidiomycota. It is saprotrophic on the dead wood of coniferous and broad-leaved trees, which are common throughout the temperate Northern Hemisphere. [9].

*F. pinicola* fruiting bodies, which are considered to be nontoxic mushrooms in Europe [10], have been used in Korean folk medicine as hemostatic and anti-inflammation agents [10, 11]. F. pinicola is known to contain a variety of primary metabolites (such as polysaccharides) and secondary metabolites (such as triterpenes, esters, lactones and steroids) [12, 13]. Extracts and isolated compounds from *F. pinicola* have demonstrated various biological activities, including antioxidant [14-16], antimicrobial [10], anti-inflammatory [17], and cytotoxic [18, 19]. Regarding the antitumor potential of *F. pinicola*, there is not much data present, but a few existing studies motivate to expand the research in this direction. Indeed, a nonspecific cytotoxic activity on human cancer cells such as HeLa and hepatocarcinoma lines SNU 185 and SNU 354 has been shown for the methanol but not for water extracts *of F. pinicola* [16].

Recently, more studies have been performed on *F. pinicola* extracts in a search for more specific anticancer activity. Chlorophorm extracts of the mushroom have demonstrated cytotoxicity, which was almost twice more specific to colorectal cancer cells (SW-480) than to control HEK293 cells. The cytotoxic effect took place through the ROS-mediated apoptotic mechanism. Moreover, the extracts were able to inhibit migration of the SW-480 cells in scratch wound and transwell assays by means of downregulation of matrix metalloproteinases [20]. The authors claim that one (but not the only) of the acting compounds of the mushroom is ergosterol, for it was one of the major components of the extracts and could produce similar, although slighter effects on SW-480 cells [20]. Another study has revealed the potential of *F. pinicola* ethanol extracts not only to induce apoptosis in various human and murine cancer cell lines, but also to significantly inhibit xenograft sarcoma-derived tumor growth in mice, along with prolongation of their survival time and absence of severe side effects, when given as a food supplement [21]. Interestingly, combined treatment of mice with the extract and a common chemotherapeutic agent cisplatin gave a synergistic effect on slowing down the tumor growth. Taken together, these findings provide a stronger evidence that apart from the unspecific cytotoxic compounds, *F. pinicola* contains substances possessing specific anti-oncogenic potential, probably acting through induction of apoptosis. Regarding the fact that *F. pinicola* is known as an anticancer agent in the Chinese folk medicine, we can conclude that this mushroom is of a certain interest for modern drug discovery as a potential source of novel anticancer compounds, which are yet to be characterized. Although ergosterol has been pinpointed as one of the candidates, the exact chemical nature of the acting compounds is still elusive. Biomolecular profiling of inedible mushrooms has revealed an unusually high phenolic content in *F. pinicola*, when compared to the others [15] and polyphenols are known to be bioactive compounds with anti-oncogenic properties [22]. Thus, detailed studies on polyphenoles of *F. pinicola* is a promising task for biomedicine.

### Hericium erinaceus

**Figure 2:**
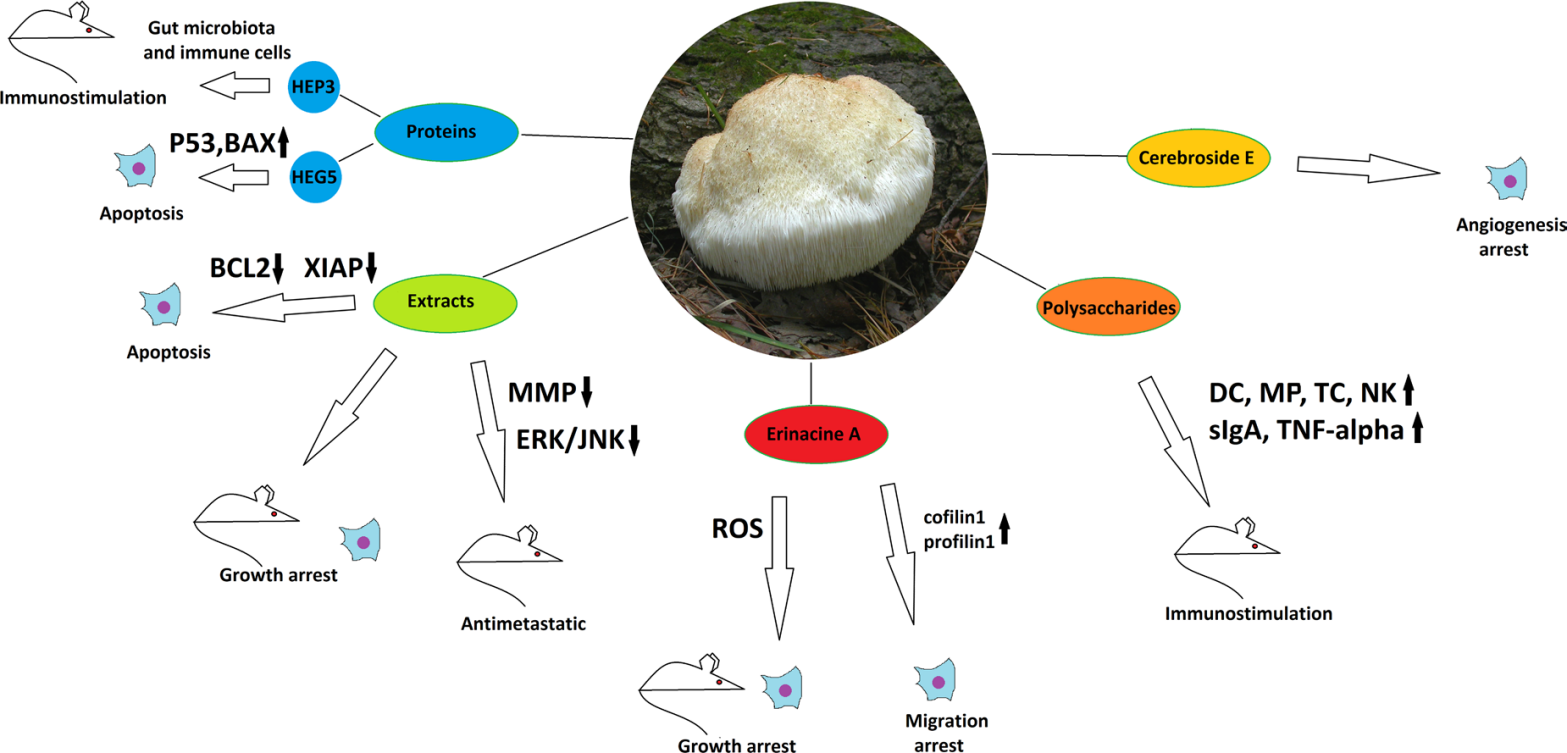
The anticancer properties of *Hericium erinaceus* Effects of different mushroom derivatives and their mechanisms of actions in various models are depicted. Mouse and cell icons indicate results obtained on animal and cell models, respectively. Arrows up and down reflect up- or down-regulation of respective proteins or pathways. ROS – reactive oxygen species, MMP - matrix metalloproteinases, DC – dendritic cells, MP – macrophages, TC – T-cells, NK – natural killers. Other proteins/pathways are mentioned under their standard names.

*Hericium erinaceus*, class Agaricomycetes, order Russulales, family Hericiaceae, is an edible medicinal mushroom (Figure 2). It is also known under the name “Lion's mane” in English, “Yamabushitake” in Japan or “ Hóutóugū ” in China. [23].

The mushroom is considered a saprotroph or a weak parasite. It is found on oak (*Quercus*) and beech (*Fagus*) in Europe, North America, Japan, Russia, and China. [24]. *H. erinaceus* has attracted special scientific attention in recent four years, being intensively studied in terms of its primary and secondary metabolites and their possible medicinal use. It yielded a number of compounds belonging to different classes with potential biological activity, which were tested against multiple targets [25-27]. Among the isolated compounds, certain were characterized, such as erinacines derived from the mycelium or hericenones derived from the fruiting bodies [27]. A significant part of research has been focused on neuroprotective properties of the mushroom, which are now extensively described in many works [28]. Another large area of possible therapeutic and anti-carcinogenic application of *H. erinaceus* is its salutary influence on the digestive organs, including stomach, liver, intestine and colon. Water and ethanol extracts of the mushroom have demonstrated growth inhibitory effects on gastric (NCI-87), liver (HepG2 and Huh-7), and colon (HT-29) cancer cell lines in the MTT proliferation assay, with the highest efficacy against Huh-7 cells (IC50 of 0.8 mg/ml for the dried extract). Although not comparing these results with non-cancer cell lines of the respective tissues, the same study describes efficient application of the extracts against xenograft tumors formed by aforementioned cancer cell lines in SCID mice. The extracts, given orally, have demonstrated a tumor suppressing activity similar to that of 5-fluoruracil, a most widely used drug clinically applied for the treatment of gastrointestinal cancers, but demonstrated a much lower general toxicity than 5-fluoruracil [29]. Another study shows that water extracts of *H. erinaceus*, given as a food supplement, possess an anti-metastatic activity, strongly inhibiting the migration of CT-26 murine colon carcinoma cells to lungs after intravenous injection into BALB/c mice, reducing the formation of tumor nodules in the lung by about 50%, and preventing metastasis-caused increase in the lung weight. The mechanism of action involves suppression of matrix melalloproteinases 2 and 9, as well as suppression of ERK and JNK kinase activation, also decreasing the general tumor cell viability [30].

Further research on tumor suppressing activity of the *H. erinaceus* extracts allowed to reveal the possible spectrum of their action modes. Studies on CT-26 derived human colon cancer xenograft tumors in mice have shown a significant reduction in tumor growth after treatment by *H. erinaceus* water extracts. It has been demonstrated that the extracts stimulated the activities of natural killer cells and macrophages on one hand and blocked angiogenesis on the other [30]. All these activities could contribute to reduction of the tumor growth, although the anticancer properties of the complex extract may not be limited by them. Another study by the same group has demonstrated a pro-apoptotic effect of same water extracts on U937 human monocytic leukemia cells in comparison to normal human and murine fibroblasts, as measured by flow cytometry. The mechanism of action is supposed to be down-regulation of anti-apoptotic proteins (Bcl-2, Bcl-xL(S), XIAP, and cIAPs), but not up-regulation of pro-apoptotic ones [31]. Further concerning the exploration of the immunomodulatory potential of *H. erinaceus*, it can be stated that polysaccharide fractions of the mushroom ethanol extract and derivatives thereof are able to promote dendritic cell maturation and dendritic cell-mediated cytokine production and T-cell proliferation [32], as well as to activate macrophages and increase TNFα production [33]. Stimulatory effects on intestinal immune system, manifested mainly through increase of surface IgA expression and natural killer cell activation, have also been reported in mouse *in vivo* experiments, when the polysaccharide fraction of *H. erinaceus* was given as a food supplement [34]. Although these investigations give no clue upon the exact structural and chemical properties of the active polysaccharides, the idea of their immunomodulatory input into the anti-carcinogenic potential of *H. erinaceus* is very attractive.

Efforts to study anticancer effects of individual compounds isolated from *H. erinaceus* have produced impressive results. Cyanthine diterpenoid Erinacine A, a mycelial derivative of *H. erinaceus*, has demonstrated growth-inhibitory activities against different cancer cell lines and tumors related to the digestive tract. It was able to arrest the cell cycle through ROS-mediated activation of the oxidative stress response, initiating potentiation of the JNK1/2 MAPKs, p70S6K and mTOR pathways in human colorectal adenocarcinoma cell line DLD-1. It also showed significant proliferation decrease of the DLD-1 and other colorectal cancer cell line HCT-116 in comparison to the normal human colonic epithelial cells when analyzed by the MTT assay. Finally, Erinacine A has demonstrated an *in vivo* inhibition of DLD-1 xenograft tumor growth in nude mice [35]. The anticancer activity of Erinacine A mediated by ROS accumulation has also been confirmed by comparative proteomic assays in human gastric cancer cell lines MKN28 and TSGH9201 [36]. Further studies have also shed light on anti-invasive properties of Erinacin A, which it demonstrated on DLD-1 and HCT-116 colorectal cancer cells in the Boyden chamber and scratch wound healing assays. Proteomic studies have revealed the actin-binding proteins cofilin-1 and profilin-1 as downstream actors activated by the Erinacine A-induced ROS response and mediating the anti-invasive effect [37]. Another class of compounds common for fungi are cerebrosides, and here cerebroside E isolated from *H. erinaceus* has shown an ability to inhibit (although slightly) the tube formation in the HUVEC cell culture, thus being a potential angiogenesis blocker. This compound also revealed antitoxic properties, reducing the damage of LLC-PK1 kidney cells after cisplatin treatment in culture – properties that may suggest it for the use in complex cancer chemotherapies [38].

Interestingly, *H. erinaceus* is not merely a source of low molecular weight biologically active compounds, but also of some proteins that possess potential tumor-suppressive activities. Indeed, a *Hericium*-derived protein HEP3, which demonstrated a complex immunomodulatory impact in mice, has also been able to strongly reduce growth of CC531 cell xenograft tumors after intraperitoneal injection. The immunomodulatory effect was induced through stimulation of the gut microbiota with the protein and involved activation of the proliferation and differentiation of T-cells and stimulation of the intestinal antigen-presenting cells [39]. Another example of bioactive protein from the same mushroom is a glycoprotein HEG-5 that was able to induce apoptosis in a gastric cancer cell line SGC-7901, stimulating the expression of pro-apoptotic factors such as p53, Bax, Caspase 8 and Caspase 3 [40].

Another possible, though indirect, activity of *H. erinaceus*, which can be relevant for the gastric cancer is the ability of the fungal extracts to remediate gastrointestinal ulcers which, on their turn, can be classified as carcinogenic conditions. *H. erinaceus* has been used to treat gastritis and gastroduodenic ulcer in the Chinese folk medicine [41]. Ethanol extracts of *H. erinaceus* have shown a cytoprotective effect after treatment of alcohol-induced gastric ulcers in rats [42]. *H. erinaceus* is also able to inhibit growth of *Helicobacter pylori*, the bacterium known to be the causative agent for gastritis and ulcer. Analyses of petroleum ether extracts of the mushroom have been performed, showing ability of the extracts to suppress the growth of six *Helicobacter pylori* strains in the microdilution assay and in the disk diffusion assay *in vitro*. Separation of the extracts yielded two active compounds, namely the 1-(5-chloro-2-hydroxyphenyl)-3-methyl-1-butanone and the 2,5-bis(methoxycarbonyl)terephthalic acid, which were responsible for the inhibitory activity [43]. A polysaccharide composed of glucose, mannose, and galactose isolated from the cultured mycelia of *H. erinaceus* [44] was able to show antioxidant properties on gastric mucosa cell line GES-1 after hydrogen peroxide treatment, which makes this polysaccharide a good candidate for one of components responsible for the mushroom's gastroprotective effect [45].

Regarding the overall anticancer potential of *Hericium erinaceus*, it can be stated that this medicinal mushroom possesses a complex of active compounds, which are able to block tumorigenesis at different stages and by different mechanisms; most of them are confirmed by both cell culture and xenograft experiments. It has been demonstrated that *H. erinaceus* extracts or fractions/components thereof exhibit: (i) immunostimulatory activities, (ii) anti-metastatic activities through inhibition of matrix metalloproteinases, (iii) gastro- and intestine-protective activities, (iv) antioxidant potential, (v) pro-apoptotic activities, (vi) inhibition of angiogenesis. This spectrum of anticancer properties is provided by different compounds: polysaccharides, lipids, terpenoids (including unique erinacines), and even proteins. Thus, two possible strategies of application of *H. erinaceus* to cancer treatment are possible: studies on the complex action of the extracts on patients with their further use as cancer-preventive food supplements, and the detailed investigation of compounds isolated from the mushroom and their mechanisms of action for using them in targeted, personalized anticancer therapy of the future. Up to date, most of the cancer-related research of the mushroom has focused on (though not limited by) gastrointestinal tumors. Many preclinical trials on tumor-bearing mice indicate *H. erinaceus* as a promising candidate for therapeutic use in this field. Nevertheless, up to date no clinical trials on the mushroom or compounds thereof exist, moreover, many active compounds are still unidentified, and many mechanisms of their action remain elusive. Thus, *Hericium erinaceus* is a relatively well-studied medicinal mushroom possessing a much larger therapeutic potential for the future compared to its currently exploited applications. Regarding the possibility to culture this mushroom on industrial scale [46], it has a great chance to become a part of modern natural products-based medicinal biotechnology.

### Inonotus obliquus

**Figure 3:**
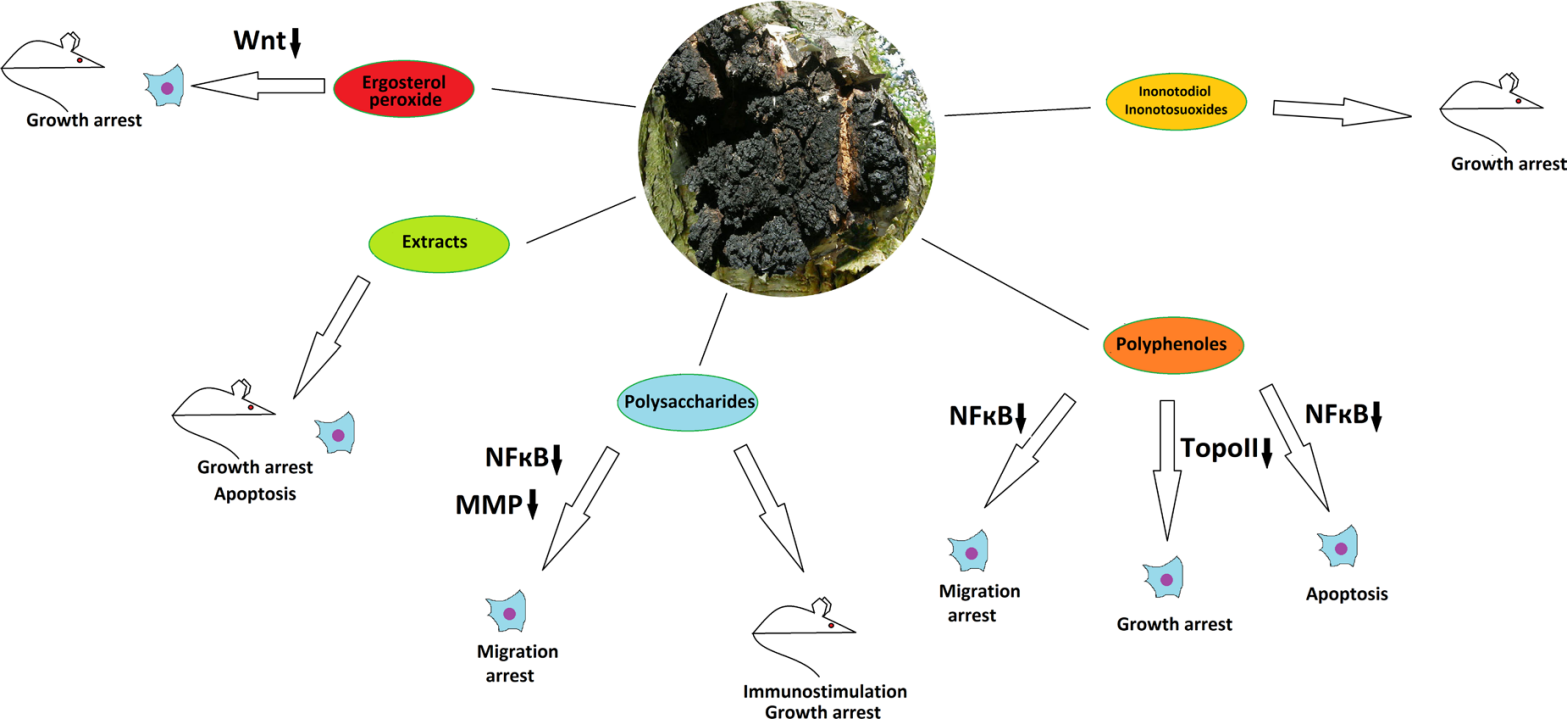
The anticancer properties of *Inonotus obliquus* Effects of different mushroom derivatives and their mechanisms of actions in various models are depicted. Mouse and cell icons indicate results obtained on animal and cell models, respectively. Arrows up and down reflect up- or down-regulation of respective proteins or pathways. ROS – reactive oxygen species, MMP - matrix metalloproteinases, TopoII – topoisomerase II. Other proteins/pathways are mentioned under their standard names.

The mushroom *Inonotus obliquus*, class Agaricomycetes, order Hymenochaetales, family Hymenochaetaceae (also known as Chaga mushroom, Figure 3) is a fungus that preferably grows as parasite on the trunks of living birch trees in the colder northern climates [47].

*I. obliquus* has long been used in folk medicine for cancer treatment in Russia, China, Korea and Japan [48-50]. Water extracts of *I. obliquus* have demonstrated cytotoxic and antimitotic activity on HeLa cells [51]. Extracts obtained from the mushroom by submerged fermentation induced apoptosis in the human colorectal carcinoma cell lines HCT-116 [52] and HT-29 [53]. Similar low-specific cytotoxic and/or cytostatic effects of the Chaga extracts were reported on human colon cancer cells [53] and liver cancer HepG2 cells [54] without, however, elucidating the mechanisms of action. *In vivo* experiments with *I. obliquus* extracts have provided data on reduction of tumor growth by induction of apoptosis in human melanoma B16-F10 cells-derived xenografts in mice [55]. Growth of human Sarcoma-180 cells-derived xenografts was as well suppressed by different sub-fractions of the Chaga extract [56].

Regarding the anticancer potential of individual compounds of *I. obliquus*, several groups can be highlighted for this mushroom. Unique lanostan-type triterpenoids inonotodiol and inonotsuoxides have revealed anti-carcinogenic effects *in vivo* using the mouse skin [48, 57] and human leukemia-derived mouse xenograft tumors [58]. Low molecular weight polyphenolic compounds demonstrated a topoisomerase II inhibiting activity leading to growth reduction in cultured human colon HCT116 carcinoma cells, identifying these polyphenoles as putative anticancer chemotherapeutic agents [59]. Another Chaga-derived polyphenol, 3,4-dihydroxybenzalacetone, inhibited the NF-κB activation and NF-κB-dependent gene expression in a panel of human cancer cell lines through blockade of IκBalpha (a subunit of NF-kappaB) phosphorylation and inhibition of NF-κB activity followed by suppression of synthesis of TNF-induced and NF-κB-dependent proliferative, anti-apoptotic and pro-metastatic gene products. These effects led to increase of TNF-induced apoptosis and decrease of TNF-induced invasion [60]. As *Hericium erinaceus*, the Chaga mushroom is extremely rich in polysachharides, which may perform immunomodulatory functions and inhibit tumorigenesis. *In vivo* trials of different Chaga-derived polysaccharides with different mouse xenograft tumor models have demonstrated reduction of tumor growth along with immunostimulatory effects [61-63]. Polysaccharides from *I. obliquus* have also demonstrated anti-metastatic activities and inhibition of migration in cancer cell culture experiments, by blocking the expression and activity of matrix metalloproteinases 2 and 9 via suppression of MAPKs, PI3K/AKT, and NF-κB signaling pathways [64, 65]. It is also important to highlight that the Chaga mushroom contains ergosterol peroxide that has been reported to inhibit growth of several human colorectal cancer cell lines and of colon tumors in a mouse model through the mechanism of Wnt/β-catenin pathway downregulation [66]. This is particularly important, because over-activation of this pathway is a cause of many cancer types, such as colon, liver and breast and, moreover, is highly specific to cancer in adult patients being virtually inactive in healthy tissues [67]. Nevertheless, it has to be pinpointed that the most recent studies confirm the Wnt/β-catenin-inhibitory properties of the Chaga mushroom but indicate other major compound as the active one, namely the inotodiol, which efficiently suppressed Wnt-dependent breast cancer proliferation under diabetic conditions in a rat model [68]. As ergosterol is also found in other medicinal mushrooms including *Fomitopsis pinicola* discussed above, it can be one of important components of targeted cancer fungotherapy in general. Recent HPLC-tandem mass-spectrometry study of Chaga mushrooms derived from France, Canada and Ukraine suggests that the Chaga of French origin is the most rich on betulin and betulinic acid, whereas Canadian Chaga is more rich on inotodiol [69]. Taken together, the Chaga mushroom can be regarded as a very promising but somewhat understudied species, because in spite of its broad use in folk medicine and of promising activities of extracts and certain compounds against cancer *in vitro* and *in vivo*, not many exact mechanisms of action are determined and no clinical trials on human patients have been performed.

### Trametes versicolor

**Figure 4:**
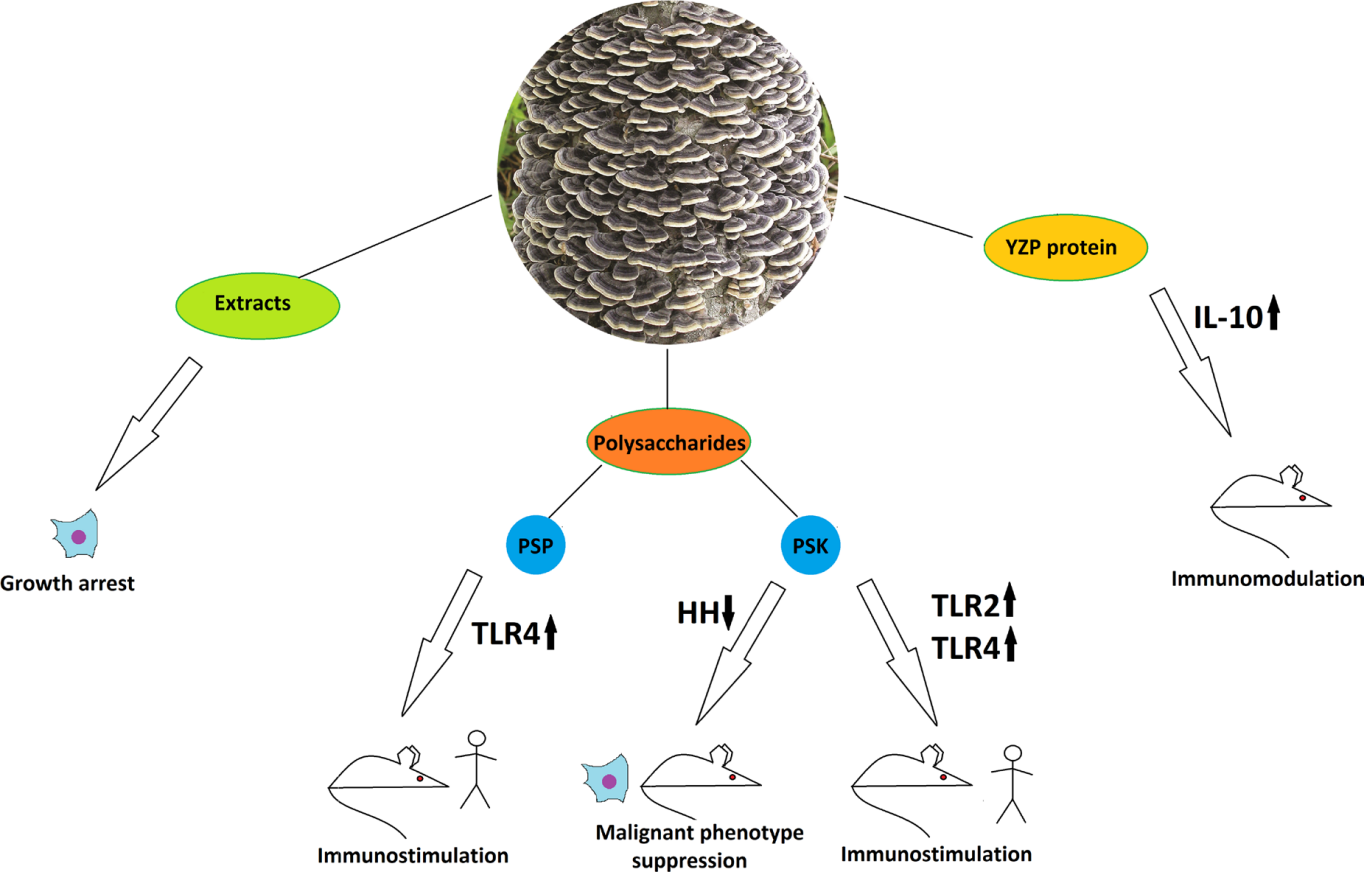
The anticancer properties of *Trametes versicolor* Effects of different mushroom derivatives and their mechanisms of actions in various models are depicted. Human, mouse and cell icons indicate results obtained in human patients, animal and cell models, respectively. Arrows up and down reflect up- or down-regulation of respective proteins or pathways. PSP – polysaccharopeptide, PSK – polysaccharide Krestin, HH – Hedgehog pathway, TLR2, TLR4 – Toll-like receptors 2 and 4. IL-10 – Interleukin 10.

*Trametes versicolor*, class Agaricomycetes, order Polyporales, family Polyporaceae (Figure 4), is a medicinal mushroom also known as *Coriolus versicolor* or *Polyporus versicolor*, “Yun-Zhi” in China, “Kawaratake” in Japan, and “Turkey tail mushroom” in English. This fungus has been used as a therapeutic agent worldwide [70]. It grows on tree trunks throughout the world in many diverse climates, including North America [71].

A solid body of data exists on *T. versicolor* cytotoxic, cytostatic and pro-apoptotic actions on various cancer cell lines. Water-ethanol extracts of the mushroom caused the proliferation inhibition on three human breast cancer cell lines (T-47D, ZR75-30 and MCF-7), human cervical cancer cell line Bcap37, human B-cell lymphoma (Raji), human promyelocytic leukemia (HL-60, NB-4), and human liver cancer cell line 7703 [72, 73]. Such results do not prove anticancer function *per se*, but provide some clues and fit well within the context of other, more detailed data. Other studies have shown anti-proliferative effects of an aqueous extract of *T. versicolor* on human breast cancer (4T1), prostate cancer (DU145), and hepatocellular carcinoma (HCC), when compared to rat normal intestinal epithelial cells (IEC-6), and African green monkey normal kidney (Vero) cell lines using the MTT assay. The results demonstrated that the *T. versicolor* extract was able to inhibit proliferation of DU145 and 4T1 cell lines in a dose-dependent way. The extract however did not exert any significant anti-proliferative effect on HCC, IEC-6, and Vero cell lines (IC_50_>1000 μg/ml), showing its selective cytotoxicity for certain types of cancer and its safety for normal cell lines. Studies of the *T. versicolor* ethanol extract effects on human prostate cancer cell lines have demonstrated the selectivity towards inhibition of growth of the androgen-responsible cell line LNcAP, while producing slight or no effect on hormone-independent lines PC-3, DU-145, and JCA-1 [74].

Separation of *T. versicolor* extracts has yielded many fractions and compounds, exhibiting targeted impact on cancer cells *in vivo* and *in vitro*. The most remarkable among them is the polysaccharide fraction which, along with some isolated carbohydrates and proteoglycans, possessed complex immunomodulatory potential similar to that of *Hericium erinaceus* and relevant for the anticancer treatment. As a recent example, a new glucan has been isolated from this mushroom by hot water extraction and subsequent chromatographic purification and has demonstrated an ability to significantly inhibit the xenograft sarcoma growth in mice [75]. Most clinically relevant representatives of *T. versicolor*-derived polysaccharides are the β-glucan-based polysaccharopeptide fraction (PSP) and the polysaccharide fraction known as Krestin (PSK) [76]. Both have underwent excessive clinical and preclinical studies as immunotherapeutic anticancer agents [77]. Immunotherapy employing PSP has already become a routine clinical practice in Japan since 1977 and in China since 1987. PSP activates cells of the immune system, boosts production of cytokines and chemokines such as TNFα, interleukins (IL-1β and IL-6), histamine, and prostaglandin E, stimulates dendritic and T-cell infiltration into tumors and reduces the harmful side effects of chemotherapy [78]. Some studies have been performed to reveal the mechanism of PSP interaction with the immune system. Experiments on peripheral blood mononuclear cells from breast cancer patients have shown that PSP drives cytokine expression through activation of the TLR4-TIRAP/MAL-MyD88 signaling pathway [79]. It has also been found out that PSP treatment leads to increased proliferation of the peripheral blood monocytes, but does not directly affect the proliferation of T, B, and NK cells [80]. Nevertheless, orally given combination of PSP with acaccia resin as an adjuvant has led to a significant increase of a hapten-induced specific T-cell dependent B-cell response in mice, suggesting a complex mechanism of PSP action [81]. The *T. versicolor*-derived polysaccharide Krestin is an even more widespread and better-studied immunomodulator used for anticancer treatment. It is able to activate different types of immune cells. PSK has shown the ability to stimulate dendritic cells through the TLR2 receptor *in vitro* and to inhibit breast cancer growth in the mouse model with the antitumor effect dependent on CD8^+^ T-cell and NK cells, but not CD4^+^ T-cells. PSK did not inhibit tumor growth in TLR2^-/-^ demonstrating that it is a specific TLR2 agonist and has potent antitumor effects via activation of both innate and adaptive immunity [82, 83]. In another study, PSK enhanced the effect of trastuzumab-mediated anti-breast cancer therapy when given orally, activating the NK cells both directly and via interleukin-12 [84]. PSK has also been able to activate murine macrophages via the TLR4 pathway, inducing TNFα and IL-6 secretion by wild type but not by TLR4-deficient peritoneal macrophages [85]. It as well potentiated docetaxel-induced tumor suppression and antitumor immune response in an immunocompetent murine model of human prostate cancer [86]. Recent studies indicate that the TLR2 agonist in PSK is a lipid component, which acts cooperatively with the protein-bound β-glucan [87].

Interestingly, a protein YZP purified from *T.versicolor* has demonstrated a related ability: specific triggering of differentiation of CD1d^+^ B cells into IL-10-producing regulatory B-cells, which promote the anti-inflammatory function [70]. It is also worth-mentioning that PSK has been shown to downregulate the over-activated Hedgehog signaling cascade under hypoxic conditions and to suppress the malignant phenotype in pancreatic cancer *in vitro* and in mouse models [88]. This may be extremely important, for Hedgehog upregulation is a well-known hallmark of many cancer types and a desired therapeutic target [89]. Here we, again, observe a complex and synergistic action of several chemically diverse compounds from the same fungal species. Nevertheless, results on clinical trials exist that do not prove high therapeutic efficacy of PSK in human patients. Indeed, in Japan, PSK has been used for adjuvant immunotherapy against gastric cancer. Patients with stage II/III gastric cancer who underwent a surgical resection were included into a retrospective study. All patients received oral fluorinated pyrimidine anti-metabolites with or without PSK after the operation, and no significant difference between the control and the PSK group in relapse free survival was detected [90]. Such examples reflect that not all data obtained in model systems are applicable to real clinical practice, and cancer therapies have to be chosen very carefully to yield the desired effects. Successful applications of PSK in human patients have been demonstrated when the polysaccharide was applied to treat lung cancer. Different sets of data on non-randomized and randomized controlled clinical trials exist that show improvement of various survival measures including median survival and 1-, 2-, and 5-year survival, improvement of immune function and reduction of tumor-associated symptoms [91]. In any case, larger and more rigorous randomized controlled trials for PSK in lung cancer patients have to be performed [91].

Alongside the lung cancer, *T. versicolor*-derived products are clinically applicable for the treatment of breast cancer [71]. Many studies and some clinical trials exist that describe the effect of the mushroom in animal models and human patients. Thus, a natural dietary supplement BreastDefend, which contains extracts from medicinal mushrooms including *T. versicolor*, inhibits proliferation and metastasis formation by the MDA-MB-231 invasive human triple-negative breast cancer cells in culture and suppresses their growth and breast-to lung cancer metastasis in a xenograft mouse model [92]. There are single-case reports [93] and Phase I clinical trial results [86] confirming that *T. versicolor*-based treatment may be used as a supplement to conventional anti-breast cancer therapy and lead to improvements of the immune status in immunocompromised breast cancer patients following standard primary oncologic treatment. The mushroom preparations are widely used in up-to-date integrative oncology and prescribed to patients on a regular basis [94].

In general, *T. versicolor* can be characterized as a medicinal mushroom, which is most actively used in modern medicine in terms of anticancer treatment compared to the other species discussed in this review. It is mostly used as an adjuvant for cancer immunotherapy, with data on clinical trials available, and has led to development of several commercial medicines, mostly acting as activators of the immune cells by the mushroom polysaccharide fractions through Toll-like receptors. Nevertheless, data on selective growth inhibition of certain cancer cell lines in culture, without any immune cells involved, suggest that there may be other specific mechanisms of action at play, besides the ones described before.

## CONCLUSIONS

The complex anticancer potential of medicinal mushrooms may be embodied not only through inhibition of certain cancer-specific processes or targeted activation of tumor-specific apoptosis, but also through indirect actions such as immunomodulation [95]. The polysaccharide-mediated antitumor immunomodulatory action seems to be rather common for many medicinal mushrooms and gives a major input into the therapeutic potential of at least three out of the four reviewed species, which is probably determined by similar carbohydrate composition and thus similar effects on the immune system of different mushrooms. Extrapolating these data, we can suppose that other, less studied, polysaccharide-rich mushroom species could possess similar or even superior immuno-stimulatory properties. Moreover, some of additional biological activities can be used for cancer prevention, diminishing the risk of tumorigenic conditions; to such activities we can attribute antioxidant, antibacterial and anti-inflammatory properties. That is why research on whole fungal extracts (sometimes reaching to the clinical trials) and even on extracts of complex mixtures of different medicinal mushrooms [96] are the important part of the given research field.

The four mushrooms reviewed in this article illustrate different stages of natural product-derived drug development. Each medicinal plant or fungus undergoes multiple stages of extraction, fractionation and purification of active compounds. At the same time these extracts, fractions and compounds are tested against different cancer models, from tumor-derived cell lines to animal models and clinical trials. Another dimension is studying the mechanisms-of-action and targets of the natural products and their derivatives. Maximum progress in all these trials brings us closer to a perfect natural drug for targeted cancer therapy. The mushroom discussed first in our review, *Fomitopsis pinicola*, is closer to the initial stages of involvement into modern cancer treatment: it is known to possess certain anticancer activities, and a set of compounds were isolated, but experiments on animal models and clinical trials are lacking, as well as precise studies on the molecular targets and signaling pathways affected by the fungus. *Inonotus obliquus* is a better-studied mushroom: here we have more data on mouse xenograft experiments and more molecular targets, including the Wnt/β-catenin pathway, a promising target for anticancer drugs of the future, but the medical relevance is still to be improved by clinical trials. *Hericium erinaceus* and especially *Trametes versicolor* are much more advanced in terms of medical applications due to their uncovered strong and complex immunomodulatory potential provided by rich polysaccharide and proteoglycan diversity. There are numerous clinical trials confirming applicability of these mushrooms and their extracts as components of modern anticancer chemotherapy. But the complex modes of action and molecular targets as well as exact structures of the active molecules from these mushrooms still have to be studied in more detail. In general, there has been a strong progress in the field of medicinal mushroom research in terms of anticancer drug development, but this work continues and much more progress still awaits us, especially in the fields of molecular targets of the medicinal mushrooms and the complex synergistic interplay of their different components.
